# Filaggrin and filaggrin 2 processing are linked together through skin aspartic acid protease activation

**DOI:** 10.1371/journal.pone.0232679

**Published:** 2020-05-21

**Authors:** Mark Donovan, Mélanie Salamito, Agnès Thomas-Collignon, Lucie Simonetti, Stephanie Desbouis, Jean-Christophe Rain, Etienne Formstecher, Dominique Bernard

**Affiliations:** 1 L’Oréal Research & Innovation, Aulnay-sous-Bois, France; 2 CNRS, Gif-sur-Yvette, France; 3 Hybrigenics Services, Evry, France; NYU Langone Medical Center, UNITED STATES

## Abstract

Skin aspartic acid protease (SASPase) is believed to be a key enzyme involved in filaggrin processing during epidermal terminal differentiation. Since little is known about the regulation of SASPase function, the aim of this study was to identify involved protein partners in the process. Yeast two hybrid analyses using SASPase as bait against a human reconstructed skin library identified that the N-terminal domain of filaggrin 2 binds to the N-terminal fragment of SASPase. This interaction was confirmed in reciprocal yeast two hybrid screens and by Surface Plasmon Resonance analyses. Immunohistochemical studies in human skin, using specific antibodies to SASPase and the N-terminal domain of filaggrin 2, showed that the two proteins partially co-localized to the stratum granulosum. *In vitro* enzymatic assays showed that the N-terminal domain of filaggrin 2 enhanced the autoactivation of SASPase to its 14 kDa active form. Taken together, the data suggest that the N-terminal domain of filaggrin 2 regulates the activation of SASPase that may be a key event upstream of filaggrin processing to natural moisturizing factors in the human epidermis.

## Introduction

Human skin is a multi-layered tissue composed of three compartments, the epidermis, the dermis and the hypodermis. The outermost of these–the epidermis–terminally differentiates to form a cornified protective and impermeable barrier to the external environment–the stratum corneum, which consists of several layers of enucleated cells known as corneoctyes and intercellular arrays of organized lipids. The corneocytes are flat polyhedral shaped cells primarily composed of intermediate filament networks surrounded by a highly cross-linked protein envelope [1–3]. The filaments are mainly composed of keratin organized into bundles by another protein known as filaggrin, which is a member of the S100 family of proteins encoded by the epidermal differentiation complex of genes found on chromosome 1 [4, 5]. The processing of filaggrin by a cascade of proteases also provides a source of free amino acids that are the main components of the natural moisturizing factors key to maintaining the stratum corneum in a hydrated state [6].

The skin aspartic acid protease (SASPase) is a specifically expressed protease in both the stratum granulosum (SG) and the stratum corneum (SC) of normal human skin, where it is believed to be involved in the control of epidermal terminal differentiation and desquamation [7].

SASPase is expressed in the epidermis as a 28 kDa proform where it is processed to its 14 kDa active form in the SG and SC of human skin. In psoriatic skin the 28 kDa proform of SASPase (SASPase28) is significantly present throughout the stratum corneum [7]. SASPase is also expressed in differentiated areas of squamous cell carcinomas but not in undifferentiated tumors [8]. Several studies have shown that SASPase has a key role in SC barrier function. Recent proteomics studies of subjects with dandruff scalp showed that there was a significant increase of SASPase in the SC in this condition [9]. Furthermore, de novo missense variants in SASPase in dogs result in aberrant filaggrin expression and an associated ichthyosis skin phenotype [10]. A transcriptomic study of lipid raft disruption in keratinocytes, evoking typical features of Atopic Dermatitis (AD), showed that SASPase was one of the most significantly down-regulated genes [11].

Transgenic knockout studies of the mouse homolog of the SASPase (ASPRV1) gene resulted in a finely wrinkled skin surface indicating that the protease is functionally important in mammalian tissue organization [12]. Transgenic mice that over-express ASPRV1 did not show evident characteristics on unchallenged skin but a delay was observed in wound closure suggesting that the enzyme is functionally important in skin tissue regeneration [13]. In addition, SASPase deficiency in hairless mice revealed a dry skin phenotype associated with a defect in filaggrin maturation suggesting that SASPase is indispensable for filaggrin processing [14]. More recent transgenic studies have provided evidence that ASPRV1 acts as a mediator in neutrophil induced inflammation in autoimmune disease [15].

Filaggrin 2 (FLG2) is one of the most recently described members of the S100 fused-type protein family [16]. The FLG2 gene encodes a 250 kDa protein and its N-terminal region contains a S100 calcium binding domain. It is expressed in the granulous layer of the epidermis where it is processed to smaller fragments by the protease calpain 1 [17]. The amino terminal domain of FLG2 is a component of cornified envelopes [18] and co-localizes with corneodesmosin [19] indicating that FLG2 plays a role in epidermal and SC adhesion.

Filaggrin (FLG) mutations are recognized as a major risk factor for AD [20] and mutations in the gene also underlie Ichthyosis vulgaris syndrome [21]. The protein levels of FLG2 are also decreased in the SC and epidermis of AD patients [22, 23], in the SC from dandruff condition [9], in a lipid raft disruption model [24] and in ichthyosis peeling skin [19, 25] suggesting that FLG2 plays an important role in epidermal homeostasis and barrier formation. In a reconstructed epidermal model where the expression of FLG2 was downregulated by lentivirus mediated shRNA interference, the levels of filaggrin processing enzymes were reduced, resulting in decreased levels of filaggrin derived free amino acids. These data demonstrated that FLG2 has a key role to play in the processing of filaggrin to NMFs in the epidermis of human skin [26].

Despite such knowledge of SASPase biology in skin, little is known about the regulation of its physiological function and auto-activation. Thus, the objective of this study was to identify proteins that bind to and regulate the function of SASPase in the epidermis of human skin. In this paper, we report, through yeast 2 hybrid (Y2H) analyses of a reconstructed epidermis cDNA library, the identification of the N-terminal domain of FLG2 (FLG2Nter) as a binding partner of SASPase. This interaction was confirmed through Surface Plasmon Resonance (SPR) analyses and their partial co-localization in the stratum granulosum of human skin was evidenced by immunohistochemistry. We also demonstrated through *in vitro* enzymatic analyses that FLG2Nter regulates the auto-activation of SASPase. Thus, a model describing the potential role of FLG2Nter in the regulation of SASPase activity and subsequent processing of FLG is proposed.

## Results

In order to obtain a deeper understanding of the molecular mechanism underlying SASPase autoactivation and to identify epidermal proteins that may be involved in the regulation of its function in skin, Y2H analyses were performed using a human reconstructed epidermis library and full length SASPase28 as bait as described in materials and methods. In a Y2H screen by cell to cell mating of more than 123 million clones, 29 positive interactions were detected. Twenty six of these clones represented sequences from filaggrin 2 (Uniprot Q5D862) with a predicted biological score (PBS) of class A (the score of highest confidence in the interaction). The selected interaction domain (SID) was identified to be between amino acids 2 and 213 in FLG2Nter (Table 1).

**Table 1:** SASPase 28 interacts with the N terminus of filaggrin 2 protein. An Y2H analysis used full length SASPase 28 as bait to screen more than 123 million clones in a random primed human reconstructed skin library. 26 out of 29 positive clones detected represented sequences from filaggrin 2 (Q5D862) with a PBS score class A and a SID domain between amino acids 2 and 213 in the N terminal domain of filaggrin 2.

Bait Uniprot accession no.	Number of clones obtained	PBS Score	Bound Protein Uniprot accession no.	Region involved in interaction (SID)
**SASPase Q53RT3**	**26**	**A**	**Filaggrin 2 Q5D862**	**2-213aa**

**Fig 1:**
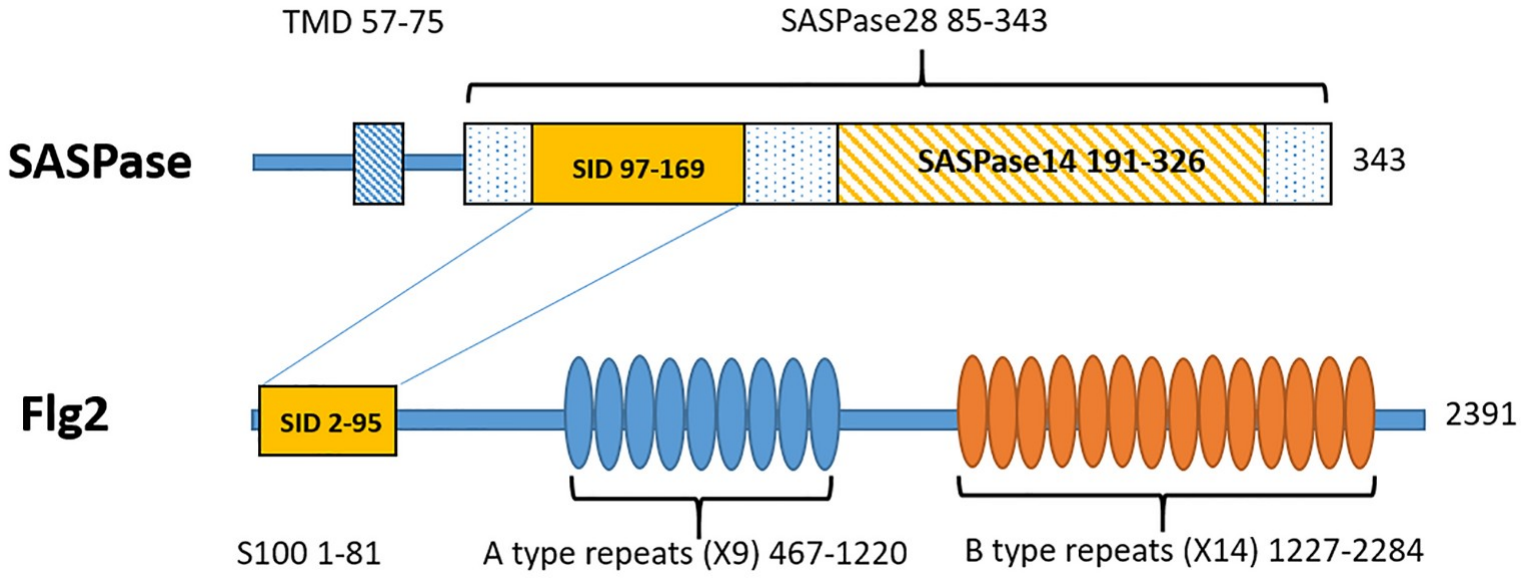
Domain organization of FLG2 and SASPase showing the single interacting domains (SID) between the two proteins.

To confirm this protein interaction, a reciprocal Y2H analysis was carried out using FLG2Nter (aa 2–213) as bait. 325 positive interactions were detected in a screen of 68 million clones in the random primed human reconstructed skin library. FLG2Nter was not an activator by itself in the Y2H assay. 307 of these clones represented sequences from SASPase (Q53RT3) with a PBS score of class A. The SID domain was identified to be between amino acids 12 and 84 in the N-terminal domain of SASPase28 (Table 2, corresponding to aa 97 to 169 in the published Q53RT3 sequence, Fig 1).

**Table 2:** Filaggrin 2 N-terminus (aa 2–213) interacts with the N-terminus of SASPase 28 protein. An Y2H analysis using filaggrin 2 N-terminus (aa 2–213) as bait was used to screen more than 68 million clones in the random primed human reconstructed skin library. 307 out of a total of 325 positive interactions represented sequences from SASPase (Q53RT3) with a PBS score class A and a SID domain between amino acids 12 and 84 in the N terminal domain of SASPase 28.

Bait Uniprot Accession no.	Number of clones obtained	PBS Score	Bound Protein Uniprot accession no.	Region involved in interaction (SID)
**Filaggrin 2 Q5D862**	**307**	**A**	**SASPase Q53RT3**	**12–84 aa(SASPase28)**

In order to determine if the S100 domain of filaggrin 2 was involved in the interaction, a third Y2H was performed using an amino acid fragment (aa 2–95) encompassing the S100 domain of filaggrin 2 as bait. In this analysis 362 positive interactions were detected out of 71 million clones screened. 355 of these clones represented sequences from SASPase (Q53RT3) with a PBS score of class A and the SID domain was identified to be between amino acids 12 and 84 in the N-terminal domain of SASPase28 (Table 3).

**Table 3:** FLG2Nter (aa 2–95) interacts with the N-terminus of SASPase 28 protein. A Y2H analysis using FLG2Nter (aa 2–95) as bait was used to screen 71 million clones in a random primed human reconstructed skin library. 355 out of 362 positive interactions represented sequences from SASPase (Q53RT3) with a PBS score class A and a SID domain between amino acids 12 and 84 in the N-terminal domain of SASPase 28.

Bait Uniprot Accession no.	Number of clones obtained	PBS Score	Bound Protein Uniprot accession no.	Region involved in interaction (SID)
**Filaggrin 2 Q5D862**	**355**	**A**	**SASPase Q53RT3**	**12–84 aa (SASPase28)**

Thus, the Y2H analyses showed that an N-terminal fragment of FLG2 (aa 2–95), which includes its S100 domain, bound to a fragment in the N-terminal domain of SASPase28 (aa 12–84) but not within the 14 kDa catalytic domain located in the C-terminus of the enzyme (Fig 1). The Y2H analyses that were performed with SASPase28 did not show interactions with the S100 proteins filaggrin, hornerin or TCHH.

In the Y2H analyses FLG2 interacted with a SID located between aa 97–169 in SASPase while SASPase interacted with a SID between aa 2–95 in the N terminal region of FLG2. The full length of SASPase is illustrated (aa 1–343) showing a putative transmembrane domain -TMD (aa 57–85), the proform of the enzyme SASPase28 (aa 85–343) and the activated form SASPase14 (aa 191–326). The full length of FLG2 is illustrated (aa 1–2391) showing the S100 calcium binding protein domain (aa 1–81), the A type repeats domain (aa 467–1220) and the B type repeats domain (aa 1227–2284).

**Fig 2:**
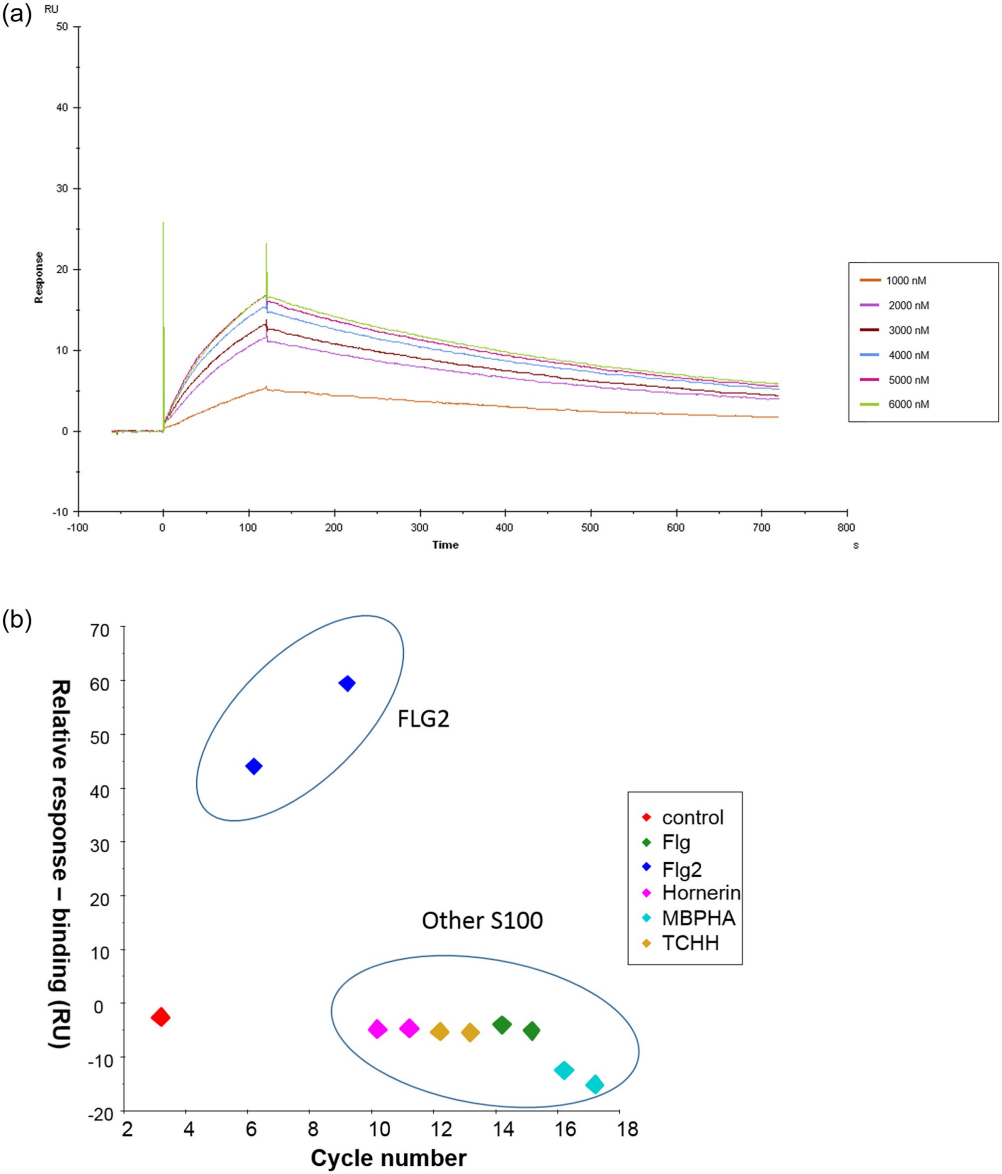
A) The N-terminal domain of Filaggrin 2 binds to SASPase 28. SPR sensorgram shows the binding between recombinant GST-Flag-SASPase 28 and MBP-HA FLG2 S100 (aa 2–95). MBP-HA FLG2 S100 (aa 2–95) was injected at 6 different concentrations (1, 2, 3, 4, 5 & 6 μM) across 1 μM of SASPase 28 captured by the GST antibody immobilized on a CM5 sensorship. B) The binding of SASPase 28 to the N terminal domain of Filaggrin 2 is specific. A goat polyclonal anti-GST was immobilized on a CM5 sensorchip to capture 1 μM of GST-Flag-SASPase 28 kDa and GST, respectively. The S100 proteins MBP-HA FLG2 S100 (aa 2–95), MBP-HA FLG S100 (aa 2–95) MBP-HA Hornerin S100 (aa 4–95) and MBP-HA TCHH S100 (aa 2–95) and the control MBP-HA were injected at a concentration of 3 and 6 μM respectively.

To confirm the association between the respective N-terminal domains of SASPase28 and FLG2Nter, the interaction in real time between the two proteins was analyzed by SPR. A goat polyclonal anti-GST immobilized on a CM5 sensorchip was used to capture a GST-Flag-SASPase 28 kDa recombinant protein and GST, respectively. A recombinant MBP-HA tagged FLG2 (aa 2–95) recombinant protein was injected onto the sensorchip at 6 different concentrations (0–6 μM). The sensorgram (Fig 2a) showed that MBP-HA FLG2 (aa 2–95) bound to GST-Flag-SASPase 28 kDa. The association of the two proteins increased in proportion to the concentration of injected MBP-HA FLG2 (aa 2–95). The binding of MBP-HA FLG2 (aa 2–95) to SASPase appeared to reach saturation at 5 μM (S2 Fig). The kinetic association (ka) and dissociation (kd) rate constants were calculated to be 4632 M^-1^s^-1^ and 2.261 10^−3^ s^-1^, respectively, yielding a dissociation constant KD of 0.488 μM. No binding was observed between MBP-HA and SASPase 28 (S2 Fig) nor was there any observed interaction between MBP-HA FLG2 (aa 2–95) and immobilized GST (S3 Fig).

Since the S100 domain of FLG2 shares significant homology to the S100 domain in the proteins filaggrin, hornerin and TCHH, a SPR binding analysis was performed, as described in materials and methods, to determine if the binding of SASPase 28 to the N-terminus of FLG2 was specific. The results showed that SASPase 28 bound the S100 domain of filaggrin 2, with a relative response binding of 60 RU, but no interaction between SASPase 28 and the S100 domains of filaggrin, hornerin or TCHH was observed (Fig 2b). Nor was there any binding observed between MBPHA and SASPase 28. Thus, the data suggest that SASPase 28 specifically binds the N terminal domain of filaggrin 2 containing the S100 domain.

The data from the Y2H and SPR analyses clearly showed a binding interaction between the N-terminal domains of SASPase 28 and FLG2 suggesting that both would be associated in the same compartments of the epidermis.

**Fig 3:**
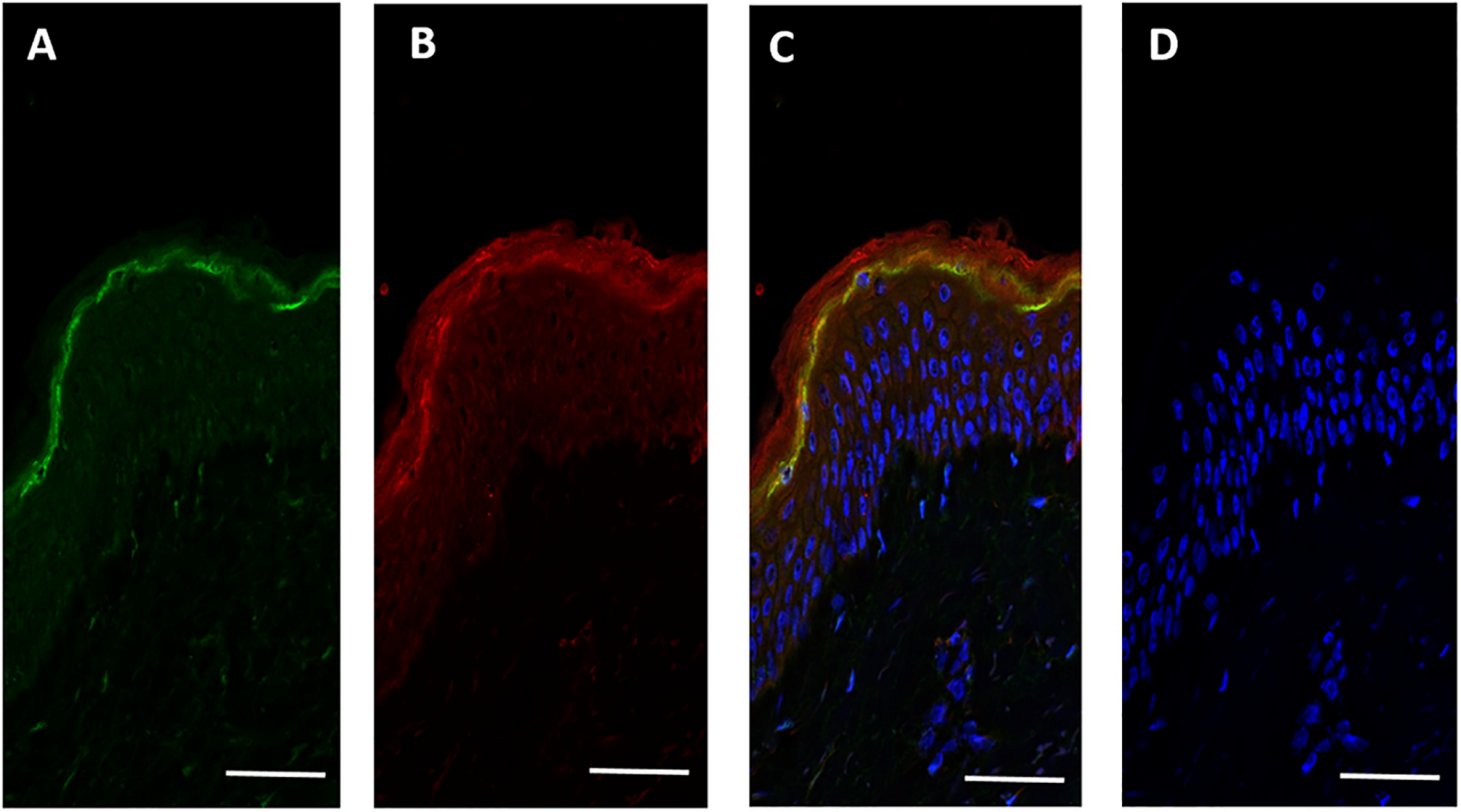
SASPase 28 and FLG2Nter co-expressed in the stratum granulosum of human epidermis. Confocal immunohistochemical analysis of the expression of SASPase 28 and the FLG2Nter on frozen sections from human skin A) SASPase 28 (in green) B) FLG2Nter (aa 2–213) (in red) C) Overlay showing co-expression of SASPase 28 and FLG2Nter in the SG of human epidermis (in yellow) D) control without primary antibodies. Nuclei stained with Hoescht dye (blue) (c & d). Scale bar = 25 μM.

In order to explore this further, a confocal immunohistochemical analysis was performed, using a recombinant antibody specific for the FLG2Nter and an affinity purified rabbit polyclonal antibody against SASPase to determine if the proteins were co-expressed within the epidermis in human skin. Both proteins were expressed and partially co-localized in the stratum granulosum of human skin (Fig 3).

**Fig 4:**
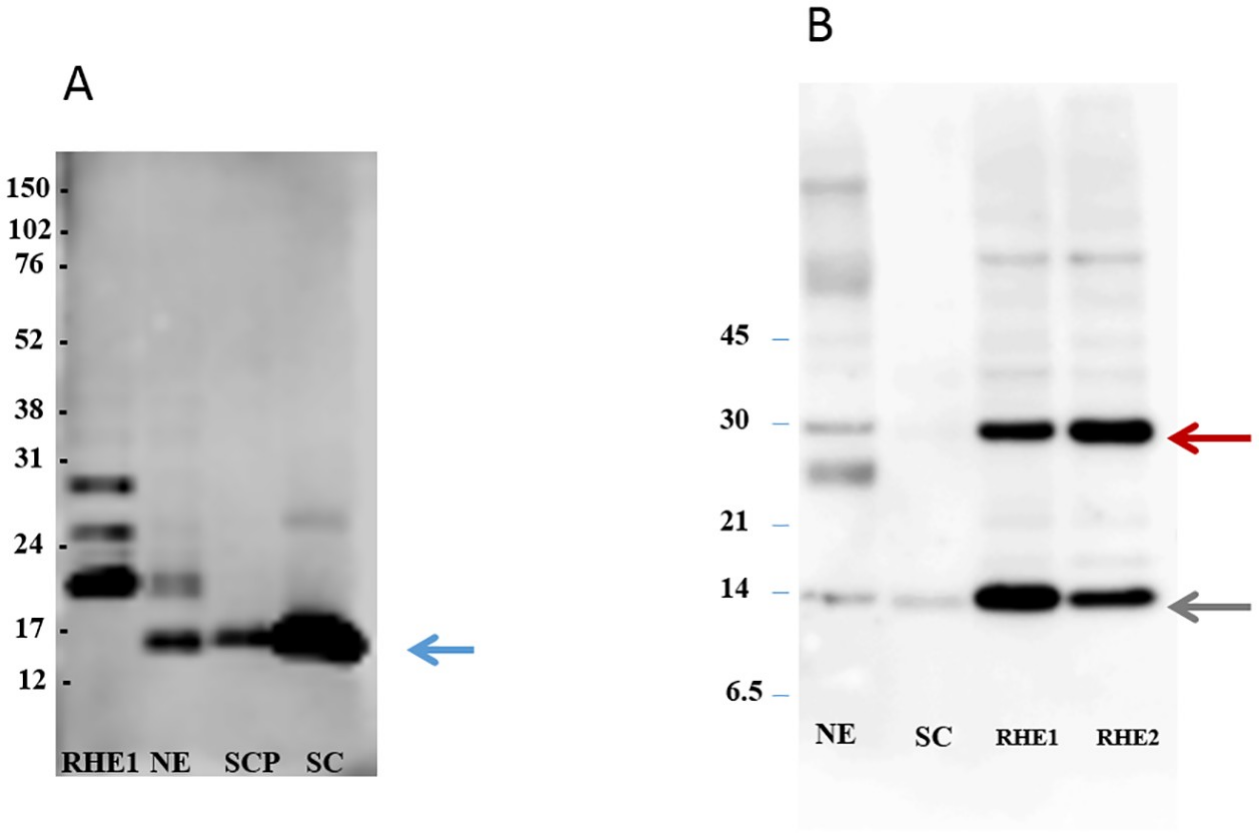
FLG2Nter is present in the stratum corneum of human skin *in vivo*. A) Western blot analysis showing the detection of a 14 kDa band (blue arrow) representing the N-terminal domain of FLG2 in soluble protein extracts from the human stratum corneum and epidermis of normal skin. The contrast was enhanced from the original image (see supplementary information) to better visualize the bands. B) Western blot analysis showing the presence of SASPase 28 (red arrow) and the 14 kDa catalytic form of SASPase (green arrow) in epidermis of human skin and reconstructed skin. (RHE = reconstructed epidermal skin; NE = epidermis from normal skin; SCP = plantar stratum corneum; SC = stratum corneum (sampled by varnish stripping). Protein Molecular Weight markers are indicated on the left of each image.

It was not clear from the immunohistochemical images in Fig 3 whether SASPase28 and FLG2Nter were also both present in the stratum corneum. Accordingly, a western blot analysis of soluble protein extracts from normal skin biopsies, reconstructed epidermal skin models, stratum corneum sampled by varnish stripping and plantar stratum corneum was performed to confirm the presence of the FLG2Nter and SASPase in the cornified layers of the epidermis. The results (Fig 4) showed that a specific recombinant antibody recognizing the FLG2Nter detected a unique and strong band at 14 kDa in human epidermis, plantar stratum corneum, and SC sampled by varnish stripping. The 14 kDa band was not detected in a reconstructed epidermis model where bands of 18, 25 and 28 kDa were detected by the recombinant antibody suggesting that in this model the maturation of the N-terminus of FLG 2 is not complete. SASPase 28 was detected in epidermal extracts from human skin and reconstructed skin models (Fig 4b—red arrow) but only the 14 kDa catalytic form of SASPase (Fig 4b—green arrow) was detected in the stratum corneum. Taken together, the data suggest that SASPase 28 was interacting with FLG2Nter in the stratum granulosum and not in the stratum corneum of human skin.

**Fig 5:**
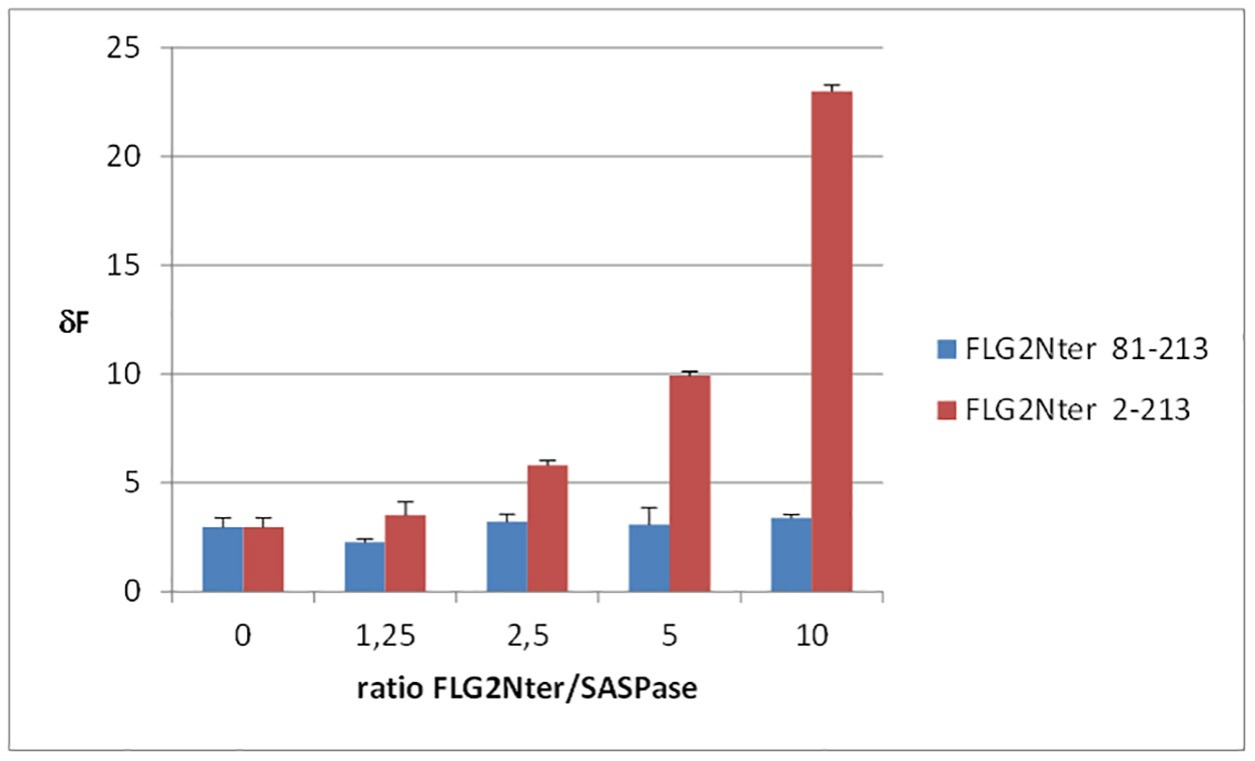
FLG2Nter activates SASPase proteolytic activity *in vitro*. An *in vitro* enzymatic assay using recombinant proteins of 28 kDa SASPase (0.5 μM), and FLG2Nter (aa 2–213) and FLG2Nter (aa 81–213) at different mass ratios to SASPase 28 in the presence of a fluorescent-labeled peptide Dabcyl-QIDRIMEK-Glu(Edans)-NH2 (0.1 mM). The histogram presents the mean values (+/-SD) of each assay performed in triplicate.

The evidence that FLG2Nter and SASPase physically interacted in the binding studies and co-localize in the epidermis and stratum corneum supports the hypothesis that the FLG2Nter may play a role in the regulation of SASPase auto-activation and activity in the differentiated layers of the epidermis. Thus, an *in vitro* enzymatic assay for SASPase 28 was carried out in the presence of FLG2Nter (aa 2–213) in order to determine if FLG2Nter could act either as an agonist or an antagonist to SASPase enzymatic activity. A quenched fluorescent tagged peptide, QIDRIMEK, which was previously identified as a preferred substrate for SASPase in a library screening assay, was incubated in a reaction buffer at pH 5.5 with the recombinant form of SASPase 28 in the presence or absence of a recombinant protein representing the FLG2Nter (aa 2–213). The results showed that the presence of FLG2Nter in a dose response manner enhanced the activity of SASPase and as much as tenfold at higher ratios as compared to the protease alone (Fig 5). Furthermore, a truncated FLG2Nter recombinant (aa 81–213) at equivalent doses did not increase the activity of SASPase in this assay (Fig 5). Thus, the results suggest that the FLG 2Nter (aa 2–213) fragment containing the S100 domain preferentially enhances the auto-activation of SASPase 28.

However, we could not conclude from this assay whether FLG 2Nter was enhancing the activity of SASPase through the stimulation of the auto activation of SASPase 28 or via a direct effect on the active form of SASPase itself.

**Fig 6:**
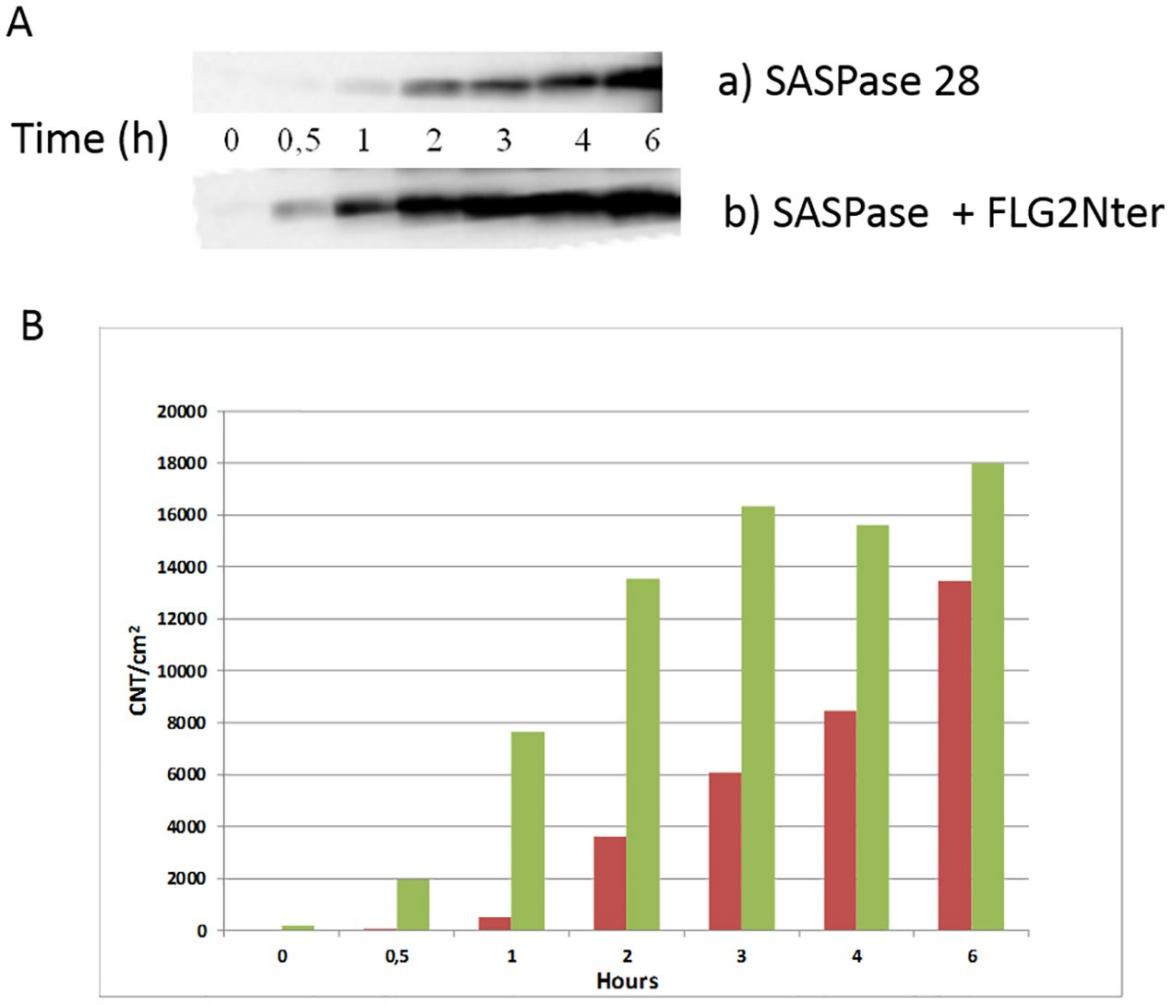
The N-terminal domain of Filaggrin 2 enhances the auto-activation of 28 kDa SASPase to its active 14 kDa form. Recombinant SASPase 28 was incubated from 0 to 6 hours in the presence of equimolar amounts of recombinant protein FLG2Nter (aa 2–213). The auto-processing of 28 kDa SASPase into its catalytic 14 kDa form was analyzed by Western blot analysis using a monoclonal antibody that detects SASPase. A cropped image from S5 Fig shows the 14 kDa catalytic of SASPase. (B) semi quantitative analysis of the SASPase 14 band in the presence of FLG2Nter (green bars) as compared to SASPase 28 alone (red bars).

Thus, in order to understand further the precise mechanism underlying the activation of SASPase the recombinant form of GST-SASPase 28 was incubated over a time course from 0 to 6 hours in a reaction buffer at pH 5.5 in the presence or absence of recombinant protein FLG2Nter (aa 2–213) at equimolar concentrations (1 μM). The auto-processing of SASPase 28 kDa to its catalytic 14 kDa form was analyzed by Western blot analysis using a monoclonal antibody which detects SASPase 14 and SASPase 28. The results showed (Fig 6 and S5 Fig) that the presence of FLG2Nter accelerated the formation of the active 14 kDa SASPase particularly after 30 minutes and 1 hour of incubation–and its presence also enhanced a greater yield of SASPase 14 as compared to the reaction where FLG2Nter was absent.

In order to address the question if FLG2Nter could directly influence the activity of the active form of SASPase we analysed the effect of FLG2Nter in an *in vitro* enzymatic assay with a recombinant protein of the active form of SASPase–SASPase 14 and the peptide Dabcyl-QIDRIMEK-Glu(Edans) as described in materials and methods. The results showed that FLG2Nter did not stimulate the activity of SASPase 14 in the assay either at equal molar concentrations (1:1) or in excess levels of FLG2 Nter to SASPase (4:1) (S4 Fig). Taken together the results from the biochemical assays in this study suggest that FLG2 Nter stimulates the activity of SASPase by preferentially stimulating its auto activation to its active form.

## Discussion

In this study we demonstrated, for the first time, through Y2H and SPR analyses, that SASPase 28 interacts with FLG2Nter. In addition, SPR studies showed that the reaction was specific to FLG2Nter among S100 related proteins. In particular no binding was evidenced to filaggrin and hornerin which share high amino acid homology in the N terminal domain to filaggrin 2. Immunohistochemical analyses showed that the two proteins were partially co-localized within the stratum granulosum of the human epidermis. FLG2Nter was also demonstrated by western blot to be present in the stratum corneum of human skin where SASPase 14 was shown to be also present in previous studies [7]. Finally, *in vitro* enzymatic assays demonstrated that FLG2Nter stimulated the autocatalytic protease activity of SASPase 28 but does not appear to modulate the activity of the catalytic form of the enzyme–SASPase 14 –once formed.

**Fig 7:**
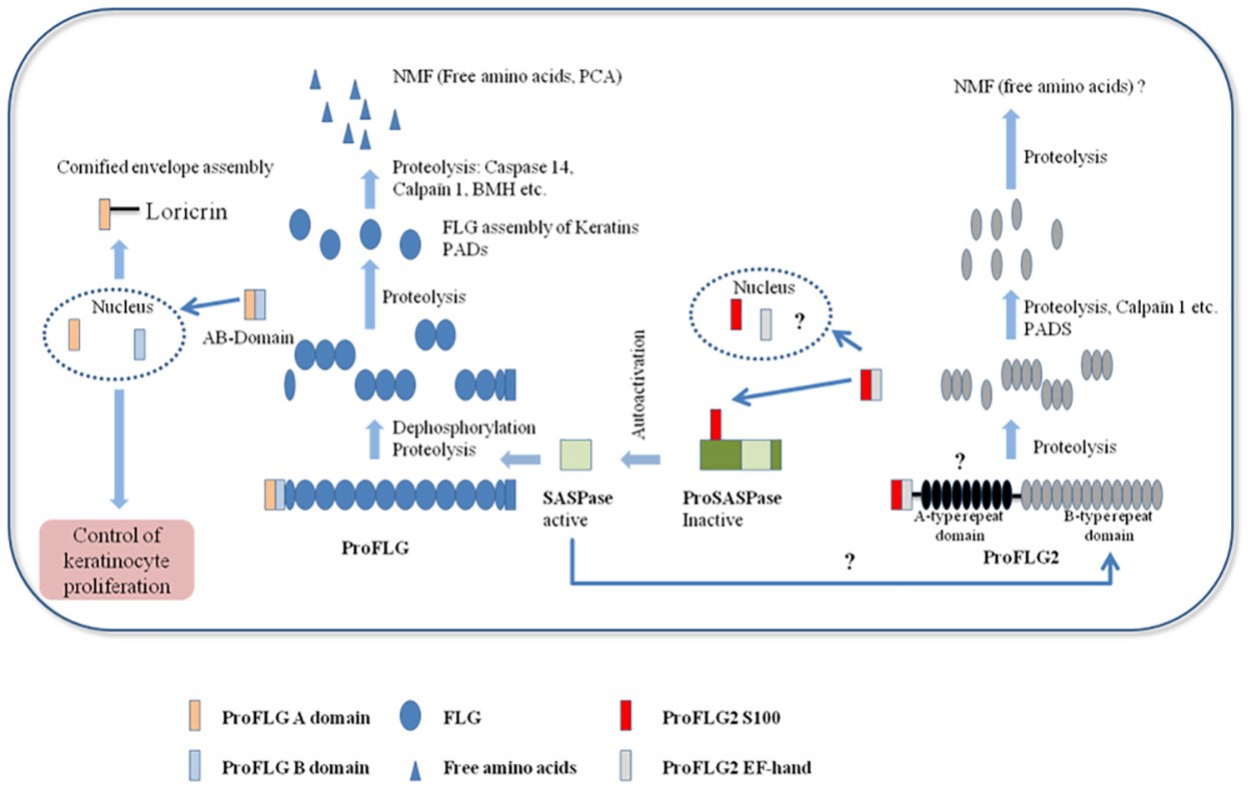
Proposed model for the role of FLG2Nter in the auto activation of SASPase and the maturation of FLG and FLG2.

Hairless mice with a deficiency in SASPase activity displayed a dry skin phenotype associated with a thicker and dehydrated stratum corneum [14]. In these mice alterations in filaggrin processing resulted in increased levels of unprocessed forms of filaggrin. Furthermore, in the same study, a recombinant protein for SASPase 14 was shown, *in vitro*, to cleave the linker sequence between the 37 kDa repeats in a recombinant filaggrin protein. Thus, the data from this study provided evidence that SASPase regulated skin hydration through the processing of profilaggrin to its intermediate isoforms. Taken together with our data, we propose a model (Fig 7) describing the role of the FLG2Nter in the auto-activation of SASPase and subsequent processing of filaggrin to its free amino acids. Since it has been shown that FLG 2 is proteolysed in part by calpain 1 [17] we propose that, during the proteolysis of FLG2 to its intermediate fragments, the N-terminal S100 domain is cleaved and binds to the N-terminal domain of SASPase 28. This interaction stimulates the auto-activation of SASPase to its active 14 kDa form, which in turn participates in the early processing of profilaggrin to its intermediate fragments, the 37 kDa filaggrin peptides and a 32 kDa profilaggrin N-terminal domain [27]. The latter fragment localizes to the nucleus and cytoplasm of epidermal granular layer cells [28] and has been postulated to play a role in signalization during epidermal differentiation [27, 29]. The hypothetical role of the proFLG N-terminal domain was partially deciphered, providing a feedback mechanism that controls epidermal homeostasis [30] and probably epidermal barrier formation through its interaction with loricrin [31]. Recently new roles for FLG2 in epidermal adhesion and barrier function have been described [18, 19, 25]. However, given the high homology between the S100 domains of filaggrin and filaggrin 2, an additional role in feedback regulation similar to that of filaggrin could also be envisaged for the FLG2 N-terminal domain, which was shown to be fully processed in the stratum corneum in this study.

In this model, FLG 2 is proteolysed, in part, by calpain 1 and other unidentified proteases, to its intermediate fragments and its N-terminal domain (FLG2Nter) [17]. The latter binds to the N-terminal domain of SASPase 28, stimulating the auto-activation of SASPase 28.

In this study, we have shown that the SASPase28 and FLG2Nter are partially co-expressed in the stratum granulosum suggesting it is in this compartment of the epidermis that FLG2Nter regulates the auto-activation of SASPase28. Since it has been shown that SASPase is one of the secreted proteases in lamellar bodies [32] and these organelles are acidic by nature [33], the modulation of SASPase auto activation by FLG2 Nter, which is optimal at an acidic pH of 5–6, may occur when the SASPase is released by lamellar bodies in the stratum granulosum.

In a reconstructed human epidermis skin model, where filaggrin 2 expression has been downregulated by shRNA technology, the proteolytic processing of filaggrin and the levels of the NMF free amino acids UCA and PCA were reduced. However, the levels of the processing protease calpain 1 were not diminished [26]. Since SASPase is a key enzyme in the early processing of filaggrin, it would be very interesting to see if the auto-activation of SASPase 28 and the consequent levels of SASPase 14 are decreased in this filaggrin 2 shRHE model. This would help explain the accumulation of profilaggrin in the SG in this model, a phenomenon, that was also observed in the SASPase -/- transgenic mouse model. If this proves to be the case it would add weight to the data suggesting that the auto-activation of SASPase is regulated by FLG2Nter.

Despite the evidence that FLG2Nter can activate the auto-activation of SASPase *in vitro*, the precise molecular mechanism through which this occurs has not been elucidated in this study. One hypothesis could be that the association between SASPase 28 and FLG2Nter leads to an allosteric conformational change of SASPase favoring a more active protein folding, similar to the mechanism through which related HIV proteases oscillate between an unfolded and folded active state [34]. Another hypothesis could be similar to the mechanism involved in the activation of cathepsin L, where auto-processing releases the N-terminal domain of the protein, which in turn, acts as an inhibitor of its activity [35]. In this scenario, auto-activation of SASPase releases its N-terminal domain which would inhibit the dimerization of the consensual aspartic protease active site [36], thus quenching activity of the enzyme. FLG2Nter could then block the inhibition of SASPase 14 dimerization by the N-terminal domain of SASPase 28 and consequently enhance the auto-activation and dimerization of the protease.

Y2H and other protein-protein interaction techniques [37] such as, co-immunoprecipitation, proximity ligation assay, enzymatic proximity labelling, or *in silico* modeling are being increasingly used to identify protein-protein interactions in order to deepen our comprehension of skin regulatory pathways. Here, Y2H was used to identify FLG2Nter as a regulator of SASPase. It was previously used to identify the interaction between FLGNter and loricrin and the its role in terminal differentiation [31]. During epidermal differentiation many proteins are processed into fragments with undefined functions, the S100 fused gene family [5], innate defense proteins like dermcidin [38], desquamation related proteases such as KLK7 [39], protease inhibitors such as LEKTI [40] or signalization protein like stratifin [41]. The identification of interacting proteins or protein domains for these released fragments would be a powerful way to decipher their role in epidermal differentiation.

In summary, the data obtained from this study showed that SASPase auto-activation is enhanced by FLG2Nter. This discovery supports the potential role of FLG2Nter in SASPase activation during the processing of filaggrin to NMFs in the human epidermis and its role in the fine tuning of epidermal terminal differentiation, SC barrier formation and skin hydration.

## Materials and methods

### Skin samples and human reconstructed epidermis

Normal human skin was obtained from surgical residues of breast reduction surgery. Stratum corneum plantar samples were obtained from foot scrapings and other stratum corneum samples were obtained from the leg by varnish stripping. All skin and stratum corneum samples were from healthy volunteers, with their written informed consent in accordance with the Declaration of Helsinki protocols and with Article L. 1243–4 of the French Public Health Code. Patients’ written informed consents were collected and kept by the acting dermatologist. The samples were anonymized before their reception by the authors. Only age, sex and anatomical site of samples were specified to the authors. The authors did not participate in sample collection. The sampling of varnish strippings were taken at an approved clinical research center (Dermexpert, Paris, France), study 07 0036 and approved by their internal ethics committee. Given its special nature, surgical residues or stratum corneum planter is subject to specific legislation included in the French Code of Public Health (anonymity, gratuity, sanitary/safety rules). This legislation does not require prior authorization by an ethics committee for sampling or use of surgical waste (http://www.ethique.sorbonne-paris-cite.fr/?q=node/1767).

Human reconstructed epidermis models were cultured until day 13 or day 20 as described [42, 43].

### Stratum corneum samplings *Varnish Stripping*

A nylon membrane (15 x 6 cm) (Millipore, Molsheim, France) with varnish solution (nitrocellulose, isopropanol, alkyd resin, acetyl tributyl citrate and ethyl acetate) was applied to the skin surface of the upper leg of volunteers—the membrane was peeled off after 10 mins. Membranes containing stratum corneum material were washed with 100 ml of ice cold acetone in a beaker. The released material, which was essentially corneocytes (cornified keratinocytes) was filtered through a 44 μm membrane. This was washed 3 times with 90 ml ice cold acetone and corneocyte material was air dried prior to extraction. Typically, 100 μl of extraction buffer was used per 1 mg of dry weight tissue.

### Yeast two hybrid analysis

The coding sequences for the full length wild type SASPase 28 (corresponding to aa 85–343 of SASPase 37), FLG2Nter (aa 2–213) and FLG2S100 (aa 2–95) were PCR amplified and cloned in frame into a pB27 plasmid as a C-terminal fusion to LexA. The constructs were used as bait to screen a random primed human reconstructed epidermis (Episkin^®^ day 13) library into the plasmid pP6. pB27 and pP6 plasmids derive from the original pBTM116 and pGADGH plasmids, respectively. More than 70 million clones were screened for each bait using a mating approach with Y187 (matα) and L40dGal4 (mata) yeast strains as previously described [43]. Positive colonies were selected on a tryptophan, leucine and histidine free medium, supplemented with 5 mM or 20 mM 3-aminotriazole for the FLG2Nter (aa 2–213) and FLG2S100 (aa 2–95) screens, respectively. The prey fragments of the positive clones were amplified by PCR and sequenced at their 5’ and 3’ junctions. The resulting sequences were used to identify the corresponding interacting proteins in the GenBank database (NCBI) using a fully automated procedure. A confidence score (PBS, for Predicted Biological Score) was attributed to each interaction as previously described [44, 45]. This allows for the ranking of the clones into classes from A (highest confidence) to D (lowest confidence) in decreasing probability of having a specific interaction with the bait. A fifth class (E) specifically flags interactions involving highly connected prey domains previously found several times in screens performed on libraries derived from the same organism. Finally, several of these highly connected domains have been confirmed as false positives of the technique and now tagged as class F. The PBS scores have been shown to positively correlate with the biological significance of interactions [46, 47]. In order to determine the interacting domains, the overlapping prey fragments from the same gene were clustered and their translated amino acid sequences aligned. The common overlapping regions were designated as the “selected interacting domain”—SID.

### Expression and purification of recombinant proteins for SASPase and FLG2Nter for SPR studies

The coding sequence for amino acids 1–259 of SASPase 28 (corresponding to amino acids 85–343 of SASPase 37) was sub-cloned into the E. Coli expression vector pEB9 with a GST-Flag tag at the N-terminus of the recombinant protein. The coding sequence containing the S100 domains (amino acids 2–95) of filaggrin 2, filaggrin, hornerin and trichohyalin (hTCHH) were sub-cloned into the E. Coli expression vector pEB7 with a MBP-HA specific double tag at its N terminus. 5 ml cultures of BL21 *E*. *coli* strains were transformed with pEB9 protein expression vector for GST-Flag-SASPase 28 and the pEB7 vectors MBP-HA-FLG2S100 (aa 2–95), MBP-HA-FLGS100 (aa 2–95), MBP-HA-HRNR S100 (aa 2–95), and MBP-HA-TCHH S100 (aa 2–95), respectively. 200 mL of culture media was inoculated with each respective transformed BL21 strain and grown at 37°C until an OD_600nm_ of 0.8–1.0. The cultures were then induced with 0.1 mM IPTG for E. Coli transfected with pEB9 GST-Flag-SASPase 28 and 0.5 mM IPTG for the pEB7 vectors and then incubated overnight at 16°C. Cells were then harvested by centrifugation; BL21 pellets were resuspended in PBS pH 7.4 supplemented with 10% glycerol and 1% Triton X-100 and lysed by sonication. Each recombinant was purified by affinity chromatography on glutathione resin for GST-Flag-SASPase 28 and amylose resin for MBP-HA-FLG2S100 and the other S100 recombinants. The elution fragments containing the fusion recombinant protein were pooled and dialyzed overnight at 4°C against PBS pH 7.4. The sample homogeneity was checked by Coomasssie blue staining of SDS-PAGE gels and the image of the gel was captured by a gel imager FluorSMax (Biorad, Marnes-la-Coquette, France) (see S1 Fig for image of GST-Flag-SASPase 28 recombinant) and its concentration was measured by Bradford assay.

### Surface plasmon resonance analysis

Binding analysis between SASPase 28 and FLG2S100 (aa 2–95) was analysed on a Biacore T200 (GE Healthcare, Orsay, France). Briefly, 1700 RU of goat polyclonal anti-GST diluted in HBS-EP+ buffer were immobilized on flow cells 1 and 2 of a CM5 sensorchip using the GST capture kit (GE Healthcare, Orsay, France). A GST recombinant protein (GE Health Care) diluted in HBS-EP+ buffer was injected into flow cell 1 at a flow rate of 30 μl/min for 180 seconds at 25°C. The ligand, GST-Flag-SASPase 28 kDa recombinant diluted in HBS-EP+ was injected into the flow cell 2 at a concentration of 1 μM at a flow rate of 30 μl/min for 180 seconds at 25°C. The analyte MBP-HA-FLG2S100 (aa 2–95) was injected at 6 different concentrations (0, 1, 2, 3 4, 5 & 6 μM) and the control recombinant MBP-HA (3 and 6 μM) into flow cell 1 and 2 at the same flow rate for 120 seconds (association) followed by injection of running buffer alone HBS-EP+ for 600 seconds (dissociation). Regeneration of the CM5 chip was performed with 10 mM Glycine-HCl at pH 2.1 for 120 seconds at 30 μl/min. Results are displayed as response units (RU) as a function of time (s). Rate equations derived from the 1:1 Langmuir binding model were fitted to the experimentally obtained association phase and dissociation phase binding curves for all injections. Kinetic association (ka) and dissociation (kd) rate constants were calculated and the KD with the formula: KD = kd/ka.

The binding analysis of between SASPase 28 and the S100 domains of the proteins filaggrin 2, filaggrin, hornerin and TCHH were also performed on a Biacore T200 (GE Healthcare, Orsay, France). Briefly, 1700 RU of goat polyclonal anti-GST diluted in HBS-EP+ buffer were immobilized on flow cells 1 and 2 of a CM5 sensorchip using the GST capture kit (GE Healthcare, Orsay, France). A GST recombinant protein (GE Health Care) diluted in HBS-EP+ buffer was injected into flow cell 1 at a flow rate of 30 μl/min for 180 seconds at 25°C. The ligand, GST-Flag-SASPase 28 kDa recombinant diluted in HBS-EP+ was injected into the flow cell 2 at a concentration of 1 μM at a flow rate of 30 μl/min for 180 seconds at 25°C. The analytes MBP-HA FLG2S100, MBP-HA FLGS100, MBP-HA Hornerin S100, MBP-HA TCHH S100 and MBP-HA were injected over both flow cells 1 and 2 at two concentrations, 3 and 6 μM, for 120 seconds at a flow rate of 30 μl/min followed by injection of running buffer alone HBS-EP+ for 600 sec (dissociation). Results are displayed as response units (RU) as a function of time (s).

### Immunofluorescence confocal microscopy

Human skin biopsies were frozen at– 80°C. Subsequent 6 μM sections were fixed with ice cold acetone for 5 min, rehydrated in PBS (Gibco^®^, Thermo Scientific, Courtaboeuf, France) and blocked with PBS/3% BSA for 20 min at RT. Sections stained for SASPase were then incubated with an affinity purified rabbit polyclonal (HPA03489 Sigma) against SASPase at 0.5 μg/ml final concentration overnight at 4 ° C. After washing for those sections stained for FLG2Nter and double staining for SASPase/FLG2Nter were incubated with a custom Alexa Fluor 546 conjugated recombinant antibody against FLG2Nter (aa 1–95)–clone AbD15810 (BioRad AbDSerotec, Puchheim, Germany) at RT for one hour–the specificity of this antibody was evaluated in ELISA assay against recombinant proteins representing the N terminal domains of filaggrin and filaggrin 2- the AbD15810 antibody only detected FLG2Nter and did not detect filaggrin. This antibody was used at a final concentration of 3 μg/ml. After washing, sections stained for SASPase and double staining for SASPase/FLG2Nter were incubated with a secondary antibody Goat anti-Rabbit IgG (H+L) Cross-Adsorbed Secondary Antibody, Alexa Fluor 488 (Invitrogen) at 2 μg/ml final concentration. All incubations (1 hour) and washes (2 x 10 min) were in PBS, 0.2% BSA at RT. Staining of sections and nuclei (Hoechst) were visualized under a Leica TCS SP8 DMI 6000 inverted microscope using a 40 X objective (Leica Microsystems, Nanterre, France).

### Expression and purification of recombinant proteins FLG2Nter for enzymatic studies

The sequence for FLG2Nter (aa 2–213) was cloned into the expression vector PEB6. Briefly, *E*.*coli* BL21 cells were transformed with the vector, cultured in LB broth at 37 ° C for 16 hours and then induced with 1 mM IPTG for 22 h at 20°C. Cells were lysed and the recombinant protein purified by Ni-NTA affinity chromatography. The quality and purification of this recombinant was verified by Coomassie stained SDS- PAGE and the image captured by a a gel imager FluorSMax in automated mode (S1B Fig).

The truncated form of FLG2Nter (aa 81–213) was cloned into a derivative of the expression vector pTFT74 and expressed and purified by the method as described [48]. Briefly, *E*.*coli* BL21 (DE3 pLys S) cells were transformed with the vector, induced with 1 mM IPTG for 3 h at 37°C and recombinant protein was purified by immobilized Ni-NTA affinity chromatography. Image of the Coomassie stained Criterion anyKD gel (Bio-Rad) was captured by a Gel Doc EZ System (Bio-Rad) using automated mode.

### Kinetic enzymatic SASPase assay

Recombinant protein representing the N-terminus of FLG2 (aa 2–213) or a truncated form of FLG2Nter (aa 81–213) was incubated in increasing concentration ratios (from 1.25 to 10) with 0.5 μM of the recombinant form of 28 kDa SASPase and 0.1 mM of a fluorescent-labeled peptide substrate Dabcyl-QIDRIMEK-Glu(Edans)-NH2 (JPT Peptide Technology, Berlin, Germany)—identified as substrate in a library screening assay—in 0.1 M acetate/150 mM NaCl pH 5.5 on a 96 well plate for 60 min at 37°C. Resulting fluorescence was then recorded (λex 340 nm, λem 490 nm) on a SpectraMax M5^e^ spectrophotometer (Molecular Devices, St Gregoire, France). To study the effect of FLG2 Nter on the catalytic form of SASPase, 1 μM of the recombinant FLG2Nter (aa 2–213) was incubated with 1 μM or 0.25 μM of a recombinant form of SASPase 14 (Abcam, Cambridge UK) and 0.1 mM of a fluorescent-labeled peptide substrate Dabcyl-QIDRIMEK-Glu(Edans)-NH2 (JPT Peptide Technology, Berlin, Germany)—in 0.1 M acetate/150 mM NaCl pH 5.5 on a 96 well plate for 60 min at 37°C. Resulting fluorescence was then recorded (λex 340 nm, λem 490 nm) on a SpectraMax M5^e^ spectrophotometer (Molecular Devices, St Gregoire, France). Each assay was performed in triplicate and the results were presented as the mean value (+/- SD).

### Auto-processing analysis of SASPase 28 kDa

The recombinant protein representing the N-terminus of FLG2 (aa 2–213) at a final concentration of 1 μM was incubated with the recombinant form of 28 kDa SASPase at a final concentration of 1 μM in 0.1 M acetate/150 mM NaCl pH 5.5 for 6 hours at 37°C. 10 μL aliquots of the reaction at time points 0.5, 1, 2, 3, 4 and 6 hours were electrophoresed on Tris-HCl 10–20% gradient SDS-PAGE gels (Biorad, Marnes-la- Coquette, France). Proteins were then transferred to PVDF membranes (Immobilon P, Millipore, Molsheim, France) under standard conditions. Membranes were incubated with anti–SASPase monoclonal antibody (clone 7H9a105) [7] followed by an anti-mouse HRP conjugated secondary antibody. Protein bands were revealed by ECL Plus^®^ (GE Healthcare, Orsay, France) and the image captured on a gel imager (FluorSmax, Biorad, Marnes-la-Coquette, France) using longer exposure (10 secs) to visualize lower molecular weight bands. The intensity of each band (CNT/cm^2^) was analyzed and integrated by Quantity One (Biorad, Marnes-la-Coquette, France).

### Western blot analysis of FLG2 and SASPase in SC and skin samples

4 mm punch skin biopsies or stratum corneum samples were homogenized in a lysis buffer of TBS/1 M NaCl/ 1% Triton X 10 containing a cocktail of protease inhibitors (Roche, Meylan, France). Lysates were clarified by centrifugation at 10 000 X g at 4°C. Protein determination was performed on subsequent soluble protein supernatants using a BCA assay (Thermo Scientific, Courtaboeuf, France) according to the manufacturer’s instructions. 10 μg aliquots of soluble protein extracts from normal skin biopsies, plantar stratum corneum (PSC) and SC obtained by varnish were separated on Tris-HCl 10–20% gradient SDS-PAGE gels (Bio-Rad, Marnes-la-Coquette, France). Proteins were then transferred to PVDF membranes (Immobilon P, Millipore, Molsheim, France) under standard conditions. Membranes were incubated with primary antibody–a His tagged recombinant monoclonal antibody (Bio-Rad AbDSerotec, Puchheim, Germany) directed against FLG2 N terminal domain (aa2-213) at 1 μg/ml followed by anti His HRP conjugated secondary antibody) (Roche, Meylan, France). For SASPase membranes were incubated with anti–SASPase monoclonal antibody (clone 7H9a105) at 1 μg/ml [7] followed by an anti-mouse HRP conjugated secondary antibody. Protein bands were revealed by ECL Plus^®^ (GE Healthcare, Orsay, France) and visualized imaged captured on a gel imager (FluorSmax, Bio-Rad, Marnes-la-Coquette, France).

## Supporting information

S1 Fig Recombinant proteins used *in vitro* enzymatic assays. Coomassie stained gels of the respective recombinants used in biochemical assays. All the images are spliced versions–as indicated by black vertical lines—adapted from the original image (see raw images file). A) GST FLAG SASPase 28 showing the recombinant migrating at 52–56 kDa–a weaker band is migrating at 25 kDa. Lane E3 is shown from the original image. B) FLG2 Nter (aa 2–213) showing the recombinant migrating at 43 kDa. Lane E5 is shown from the original image. C) FLG2 Nter (aa 81–213) showing the recombinant at 16 kDa and the fusion N1-FLG2 Nter (aa 81–213) at 25 kDa. The other bands in this purification are Sly D (35 kDa) a known His–rich E.coli protein often co purified with AgX and N1 at 10 kDa. Lane 13 is shown from the original image.
(TIF)

S2 Fig The binding of SASPase 28 to the N terminal domain of filaggrin 2 is dose dependent. A goat polyclonal anti-GST was immobilized on a CM5 sensorchip and used to capture GST-Flag-SASPase 28 kDa. MBP-HA FLG2 S100 (aa 2–95) was injected at 6 different concentrations (1, 2, 3, 4, 5 & 6 μM) across immobilized SASPase 28 on a CM5 sensorchip. The control recombinant MBP-HA was injected at a concentration of 3 and 6 μM. The graph shows the relative binding response of MBP-HA FLG2 S100 (aa 2–95) and MBP-HA to SASPase 28 kDa.
(TIF)

S3 Fig No binding was observed between GST and the N terminal domain of filaggrin 2. A goat polyclonal anti-GST was immobilized on a CM5 sensorchip and used to capture GST. MBP-HA FLG2 S100 (aa 2–95) was injected at 6 different concentrations (1, 2, 3, 4, 5 & 6 μM) across immobilized GST on a CM5 sensorchip. The sensorgram showed no observed associated or dissociated binding curves between GST and MBP-HA FLG2 S100 (aa 2–95).
(TIF)

S4 Fig FLG2Nter does not activate SASPase14 proteolytic activity *in vitro*. An *in vitro* enzymatic assay using recombinant proteins of 14 kDa SASPase and FLG2Nter (aa 2–213) at either equal mass ratios (1 μM: 1 μM) and at a ratio of 1:4 (0.25 μM: 1 μM) respectively in the presence of a fluorescent-labeled peptide Dabcyl-QIDRIMEK-Glu(Edans)-NH2 (0.1 mM). The histogram shows the relative change in activity at 30 mins of the reaction and presents the mean values (+/-SD) of each assay performed in triplicate.
(TIF)

S5 Fig Western blot larger view (cropped image from original raw image). The N-terminal domain of Filaggrin 2 enhances the auto-activation of 28 kDa SASPase to its active 14 kDa form. Recombinant SASPase 28 was incubated from 0 to 6 hours in the presence of equimolar amounts of recombinant protein FLG2Nter (aa 2–213). The auto-processing of 28 kDa SASPase into its catalytic 14 kDa form was analyzed by Western blot analysis using a monoclonal antibody that detects both forms of SASPase. Results showed that the presence of FLG2Nter accelerated the formation of SASPase 14 (indicated by blue arrow) as early as 30 minutes of incubation. The size of the GST–SASPase 28 is 52–56 kDa indicated by a red arrow–the visible bands observed between 52–56 kDa and 14 kDa are likely to be intermediate forms of the processed GST- SASPase recombinant.
(TIF)

S1 Raw images (PDF)

S1 File (DOCX)
