# Supplementary figures and images for: Filaggrin and filaggrin 2 processing are linked together through skin aspartic acid protease activation
Source: PLoS One. 2020 May 21;15(5):e0232679. doi: 10.1371/journal.pone.0232679 (PMC7241785; doi:10.1371/journal.pone.0232679)

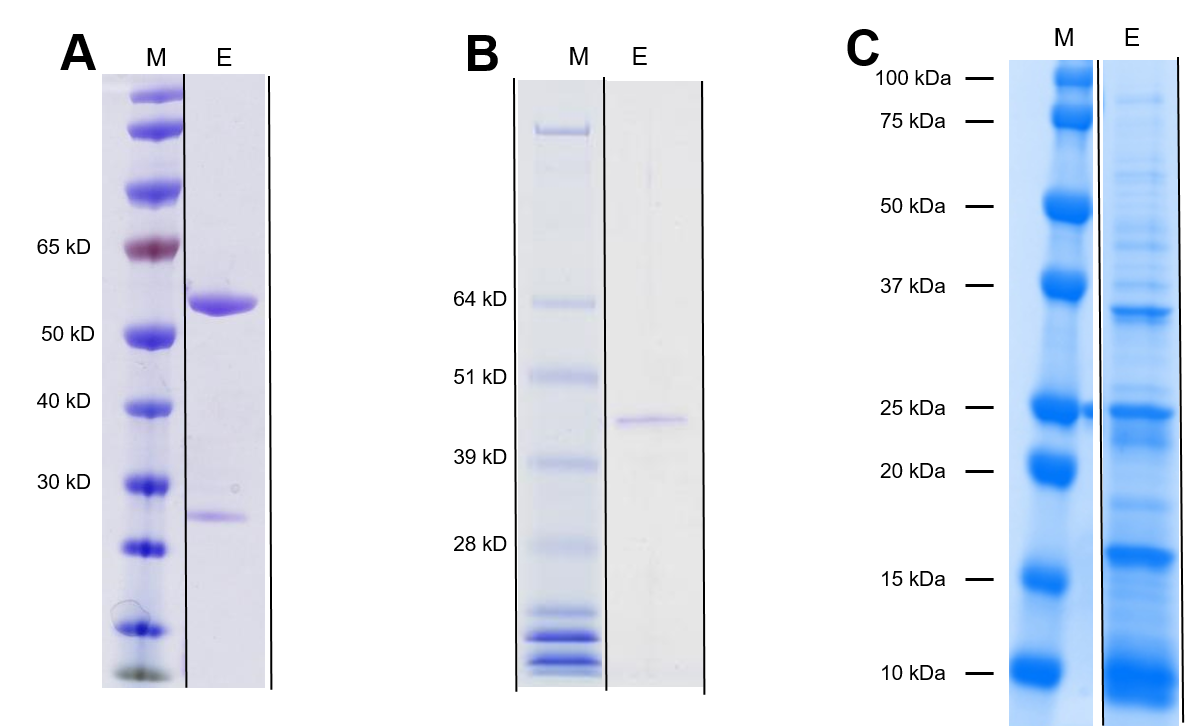

Supplement: S1 Fig — Coomassie stained gels of the respective recombinants used in biochemical assays. All the images are spliced versions–as indicated by black vertical lines—adapted from the original image (see raw images file). A) GST FLAG SASPase 28 showing the recombinant migrating at 52–56 kDa–a weaker band is migrating at 25 kDa. Lane E3 is shown from the original image. B) FLG2 Nter (aa 2–213) showing the recombinant migrating at 43 kDa. Lane E5 is shown from the original image. C) FLG2 Nter (aa 81–213) showing the recombinant at 16 kDa and the fusion N1-FLG2 Nter (aa 81–213) at 25 kDa. The other bands in this purification are Sly D (35 kDa) a known His–rich E.coli protein often co purified with AgX and N1 at 10 kDa. Lane 13 is shown from the original image. (TIF) [file pone.0232679.s001.tif]

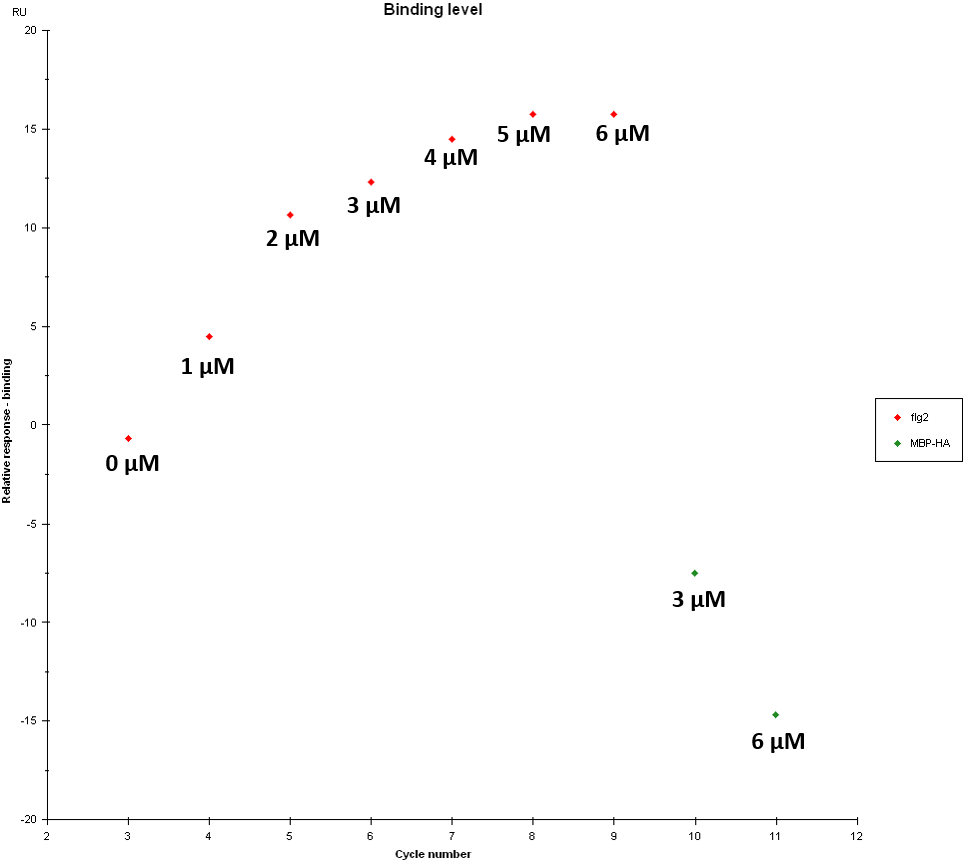

Supplement: S2 Fig — A goat polyclonal anti-GST was immobilized on a CM5 sensorchip and used to capture GST-Flag-SASPase 28 kDa. MBP-HA FLG2 S100 (aa 2–95) was injected at 6 different concentrations (1, 2, 3, 4, 5 & 6 μM) across immobilized SASPase 28 on a CM5 sensorchip. The control recombinant MBP-HA was injected at a concentration of 3 and 6 μM. The graph shows the relative binding response of MBP-HA FLG2 S100 (aa 2–95) and MBP-HA to SASPase 28 kDa. (TIF) [file pone.0232679.s002.tif]

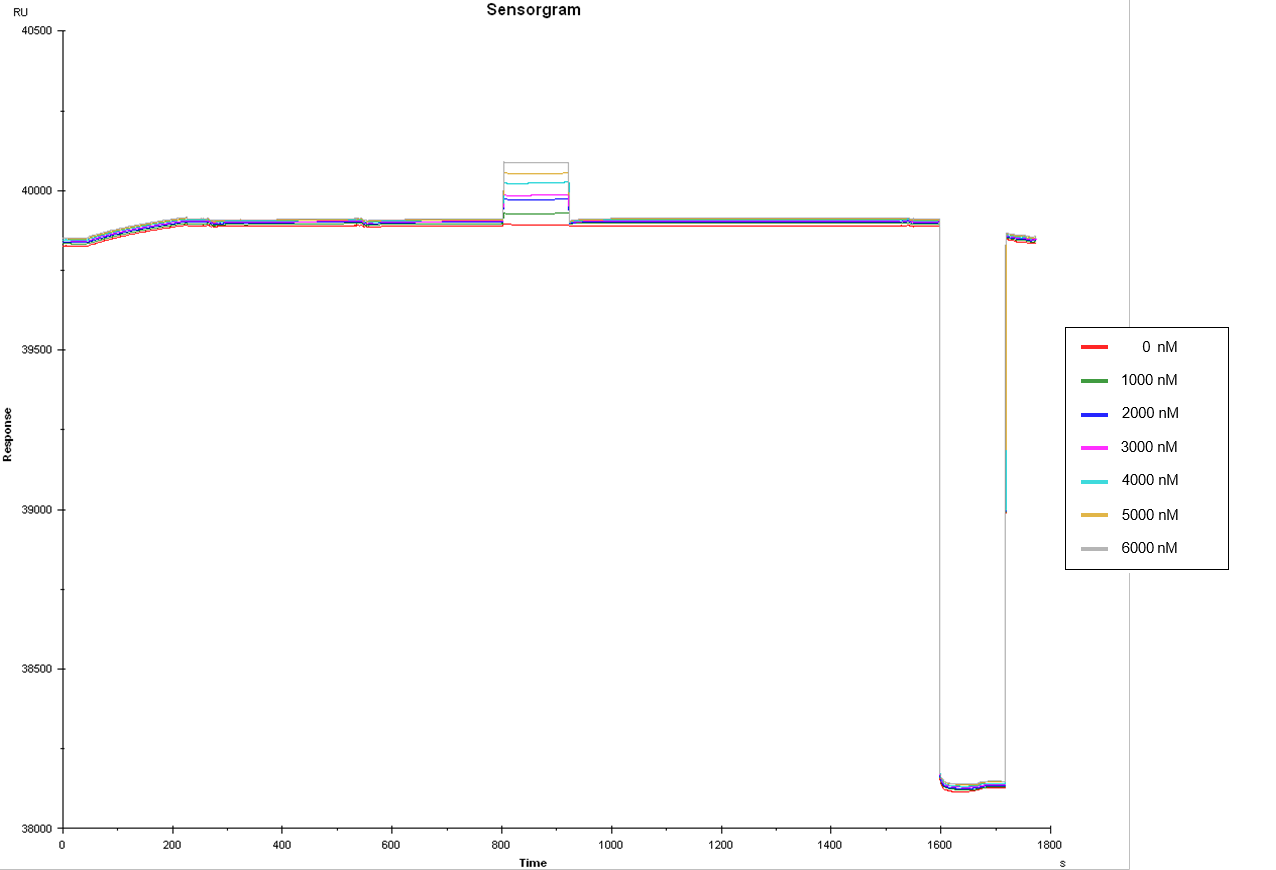

Supplement: S3 Fig — A goat polyclonal anti-GST was immobilized on a CM5 sensorchip and used to capture GST. MBP-HA FLG2 S100 (aa 2–95) was injected at 6 different concentrations (1, 2, 3, 4, 5 & 6 μM) across immobilized GST on a CM5 sensorchip. The sensorgram showed no observed associated or dissociated binding curves between GST and MBP-HA FLG2 S100 (aa 2–95). (TIF) [file pone.0232679.s003.tif]

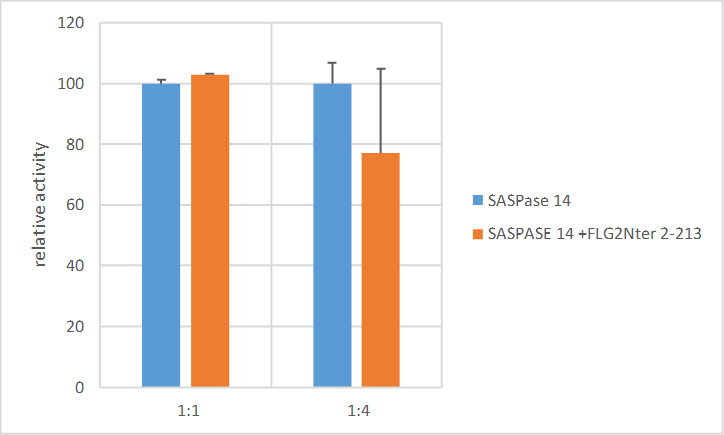

Supplement: S4 Fig — An in vitro enzymatic assay using recombinant proteins of 14 kDa SASPase and FLG2Nter (aa 2–213) at either equal mass ratios (1 μM: 1 μM) and at a ratio of 1:4 (0.25 μM: 1 μM) respectively in the presence of a fluorescent-labeled peptide Dabcyl-QIDRIMEK-Glu(Edans)-NH2 (0.1 mM). The histogram shows the relative change in activity at 30 mins of the reaction and presents the mean values (+/-SD) of each assay performed in triplicate. (TIF) [file pone.0232679.s004.tif]

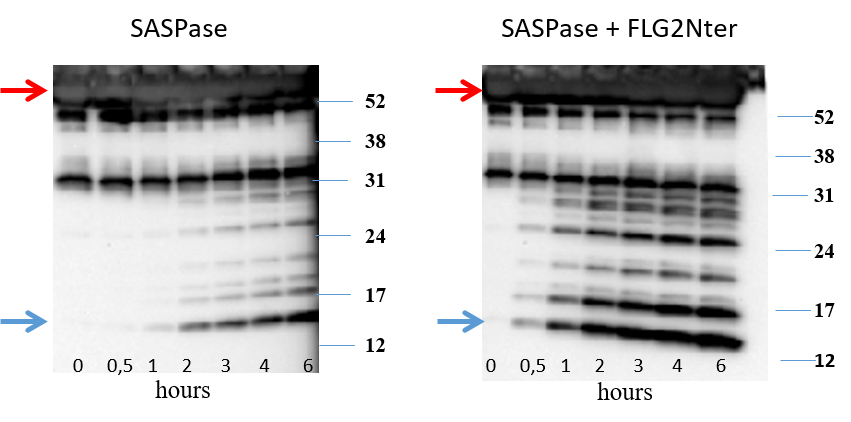

Supplement: S5 Fig — The N-terminal domain of Filaggrin 2 enhances the auto-activation of 28 kDa SASPase to its active 14 kDa form. Recombinant SASPase 28 was incubated from 0 to 6 hours in the presence of equimolar amounts of recombinant protein FLG2Nter (aa 2–213). The auto-processing of 28 kDa SASPase into its catalytic 14 kDa form was analyzed by Western blot analysis using a monoclonal antibody that detects both forms of SASPase. Results showed that the presence of FLG2Nter accelerated the formation of SASPase 14 (indicated by blue arrow) as early as 30 minutes of incubation. The size of the GST–SASPase 28 is 52–56 kDa indicated by a red arrow–the visible bands observed between 52–56 kDa and 14 kDa are likely to be intermediate forms of the processed GST- SASPase recombinant. (TIF) [file pone.0232679.s005.tif]

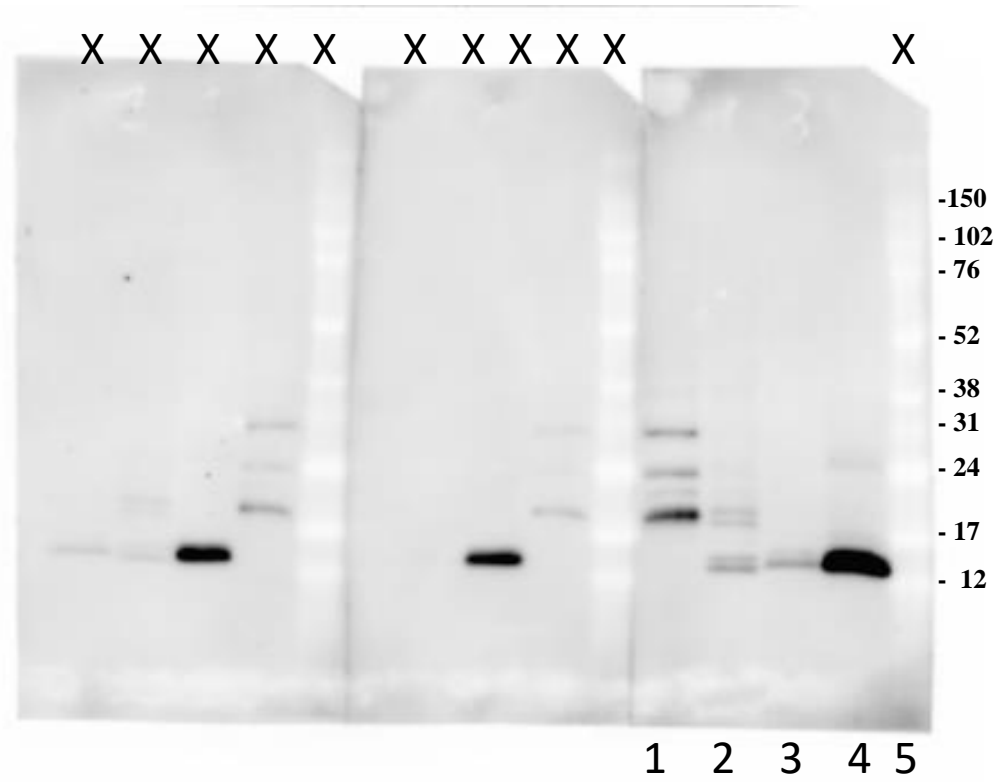

Figure 4 A

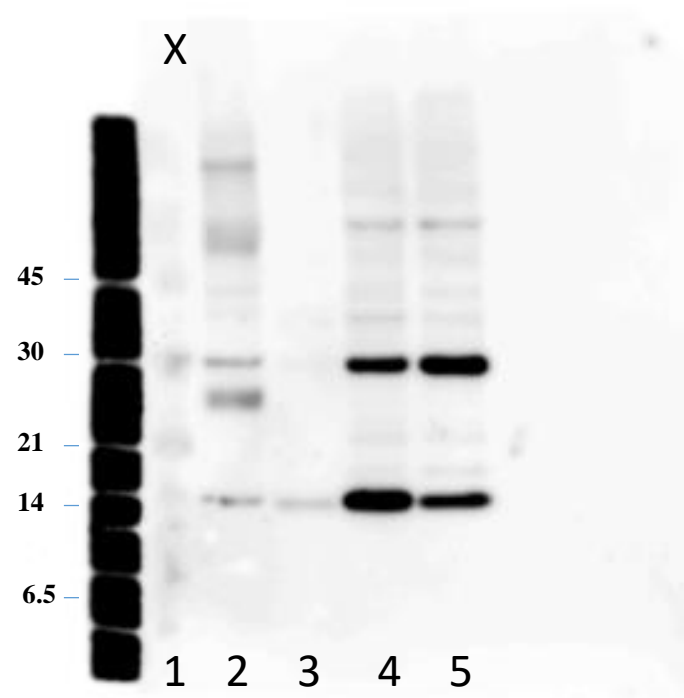

Figure 4 B

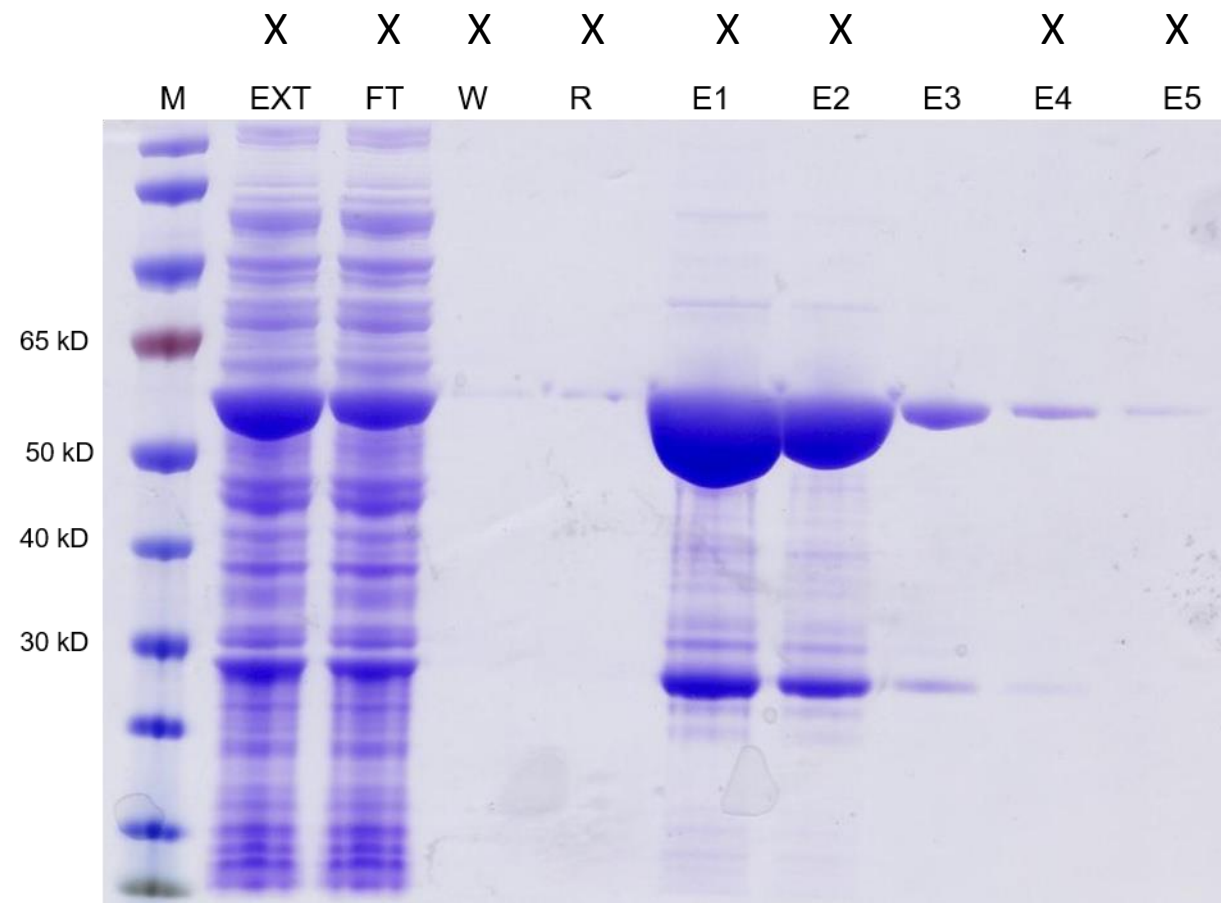

S1 Figure A

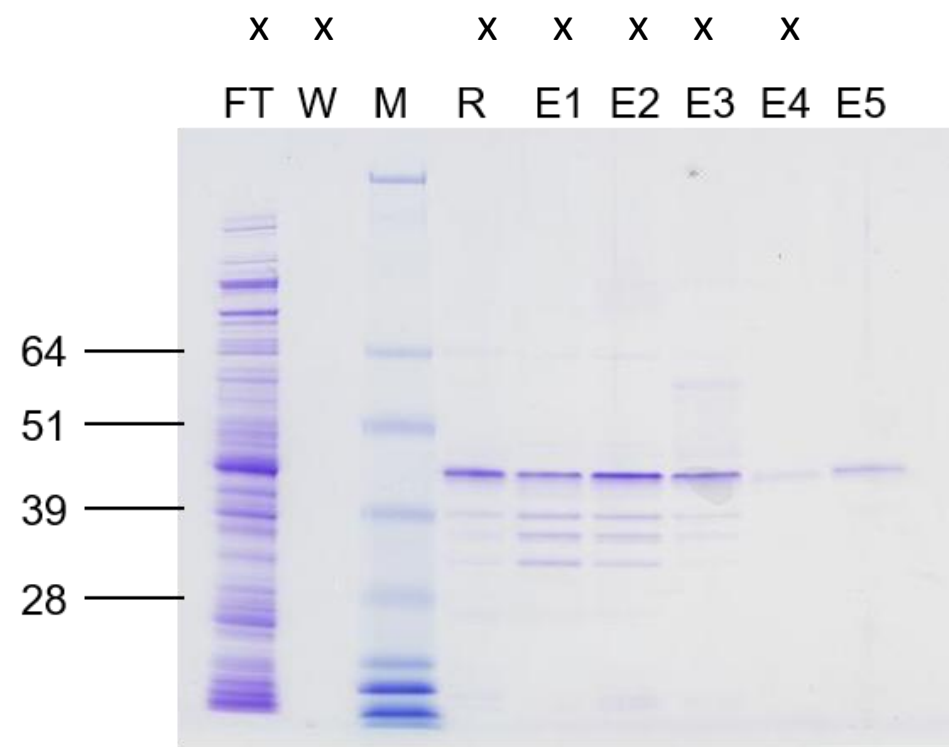

S1 Figure B

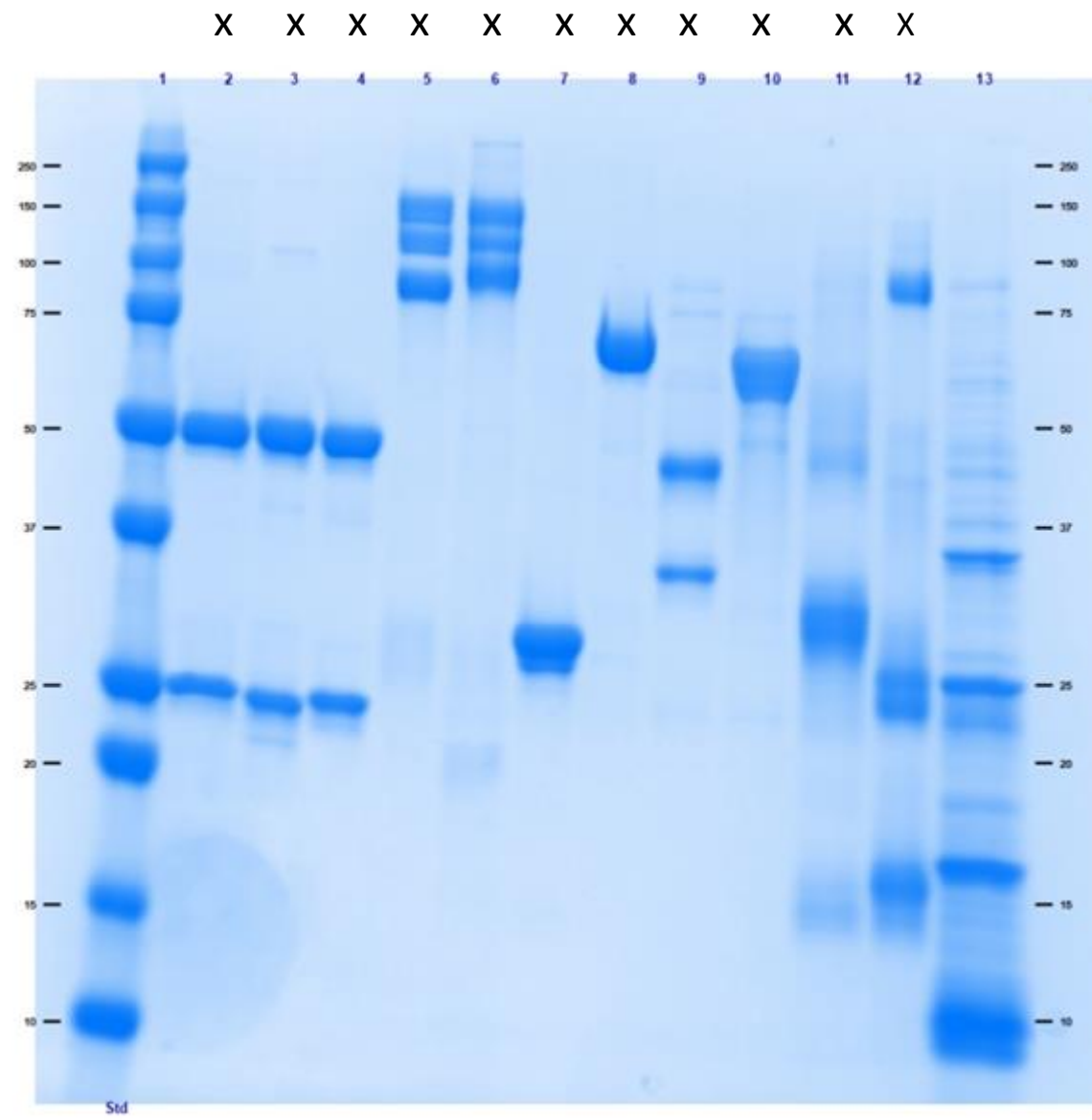

S1 Figure C

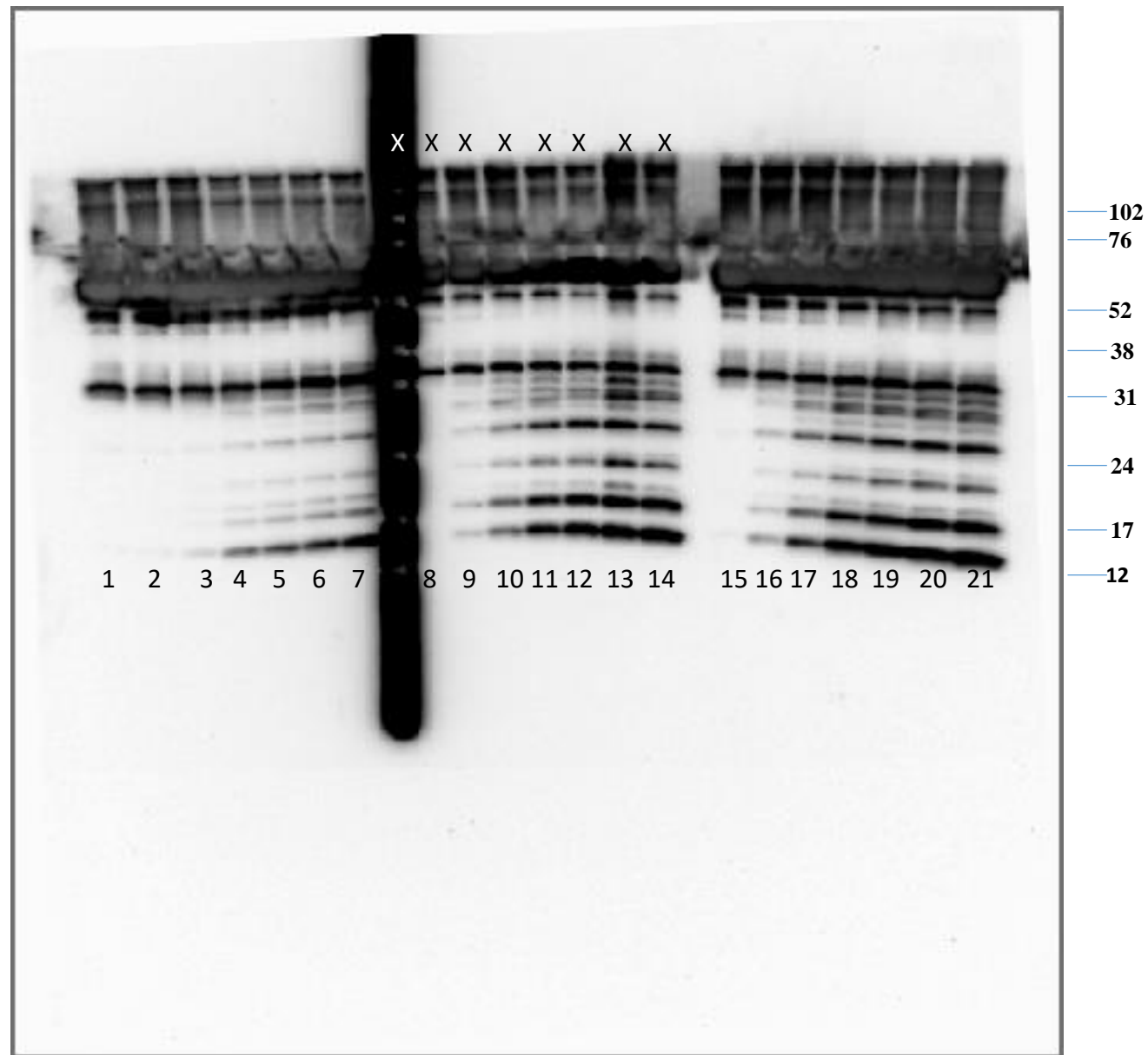

S5 Figure

Supplement: S1 Raw images — (PDF) [file pone.0232679.s006.pdf]
