# Supplementary material for: Filaggrin and filaggrin 2 processing are linked together through skin aspartic acid protease activation
Source: PLoS One. 2020 May 21;15(5):e0232679. doi: 10.1371/journal.pone.0232679 (PMC7241785; doi:10.1371/journal.pone.0232679)
Supplement: S1 File — (DOCX) [file pone.0232679.s007.docx]

**Filaggrin and filaggrin 2 processing are linked together through skin aspartic acid protease activation**

Mark Donovan^1*^, Mélanie Salamito^1^, Agnès Thomas-Collignon^3^, Lucie Simonetti^1^, Stephanie Desbouis^1^, Jean-Christophe Rain^2^, Etienne Formstecher^2^ and Dominique Bernard^1^

***^1^****L’Oréal Research & Innovation, Aulnay-Sous-Bois, France,* ***^2^****Hybrigenics Services, Evry, France,*

*^3^ CNRS Gif-sur-Yvette France*

Supplementary information on original images for gels and western blots

**Original images for Figure 4.** **FLG2Nter is present in the Stratum Corneum of human skin in vivo.**

Figure 4 Part A) Western blot analysis showing the detection of representing the N-terminal domain of FLG2 in soluble protein extracts from the human stratum corneum and epidermis of normal skin. The image was captured by a gel imager using automated mode (FluorSmax, Bio-Rad, Marnes-la-Coquette, France). The western blot on the right-hand side of the original was used for the figure with the marker lane (lane 5) cropped from the image. The contrast of the original image was increased to prepare figure 4a. Lanes 1 – 4 correspond to RHE = reconstructed epidermal skin; NE = epidermis from normal skin; SCP = plantar stratum corneum; SC = stratum corneum (sampled by varnish stripping), respectively. Lanes not used in the image are annotated by an X.

Figure 4 Part B) Western blot analysis showing the presence of SASPase 28 and the 14 kDa catalytic form of SASPase in the epidermis of human skin and reconstructed skin. The image was captured by a gel imager using automated mode (FluorSmax, Bio-Rad, Marnes-la-Coquette, France). The marker lane (lane 1) was cropped from the image. Lanes 2-5 correspond to (NE = epidermis from normal skin; SC = stratum corneum (sampled by varnish stripping). RHE1 & RHE2 = reconstructed epidermal skin, respectively.

**S1 Figure. Recombinant proteins used *in vitro* enzymatic assays.** Coomassie or equivalent stained gels of the respective recombinants used in biochemical assays.

S1 Figure Part A) GST FLAG SASPase 28 showing the recombinant migrating at 52-56 kDa – a weaker band is migrating at 25 kDa. Image of Coomassie stained SDS-PAGE gel captured by a FluorSmax gel imager using automated mode. The annotated lanes are as follows: M (protein standards- sizes indicated on left of the image); EXT (soluble protein fraction); FT (flow through non-bound proteins); W (wash fractions); R (fraction retained on resin) and E1 –E5 ( elution fractions). Lanes M and E3 were cropped out and used to generate S1Figure A. Lanes not used in the figure are annotated by an X.

S1 Figure Part B) FLG2 Nter (aa 2-213) showing the recombinant migrating at 43 kDa. Image of Coomassie stained SDS-PAGE gel captured by a FluorSmax gel imager using automated mode. The lanes annotated are as follows: FT (flow through -non-bound proteins); W (wash fractions); M (protein standards -sizes indicated on left of the image) R (fraction retained on resin) E1 –E5 (elution fractions). Lanes M and E5 were cropped out and used to generate S1Figure B. Lanes not used in the figure are annotated by an X.

SI Figure Part C) FLG2 Nter (aa 81 -213) showing the recombinant at 16 kDa and the fusion N1-FLG2 Nter (aa 81-213) at 25 kDa. The other bands in this purification are Sly D (35 kDa) a known His –rich E.coli protein often co purified with AgX and N1 at 10 kDa. Image of Coomassie stained Criterion anyKD gel (Bio-Rad) captured by Gel Doc EZ System (Bio-Rad) using automated mode. Lanes 1 and 13 were cropped out and used to generate S1Figure C. Lanes, which show other recombinant proteins not related to this study, and not used in the figure, are annotated by an X.

**S5 Figure. Western blot of** **The N-terminal domain of Filaggrin 2 enhances the auto-activation of 28 kDa SASPase to its active 14 kDa form.** Original image used to generate S5 Figure and Figure 6 part A. For enhanced imaging to visualize lower molecular weight bands ECL plus was used instead of standard ECL to detect bands. The western blot image was captured by a FluorSmax gel imager using a longer intense mode (10 secs) (Bio-Rad, Marnes-la-Coquette, France). A cropped image of Lanes 1-7 and 15-21 was used to generate S5 figure and this image was cropped to show bands indicated by the blue arrow in Figure 6A.

Lanes not used in the two figures are annotated by an X.
